# Cigarette smoking decreases macrophage-dependent clearance to impact the biological effects of occupational and environmental particle exposures

**DOI:** 10.3389/fpubh.2025.1558723

**Published:** 2025-04-09

**Authors:** Andrew J. Ghio, Matthew Stewart, Rahul G. Sangani, Elizabeth N. Pavlisko, Victor L. Roggli

**Affiliations:** ^1^US Environmental Protection Agency, Research Triangle Park, NC, United States; ^2^Department of Environmental Health Engineering, Johns Hopkins Bloomberg School of Public Health, Baltimore, MD, United States; ^3^Department of Medicine, West Virginia University, Morgantown, WV, United States; ^4^Department of Pathology, Duke University Medical Center, Durham, NC, United States

**Keywords:** smoking, silicosis, anthracosis, asbestosis, occupational exposures, environmental exposures

## Abstract

The retention of occupational and environmental particles in the lung is a primary determinant of biological effects. In the distal respiratory tract, particle clearance includes phagocytosis by alveolar macrophages (AMs), migration to the terminal bronchiole, and transport of AMs and particles by the mucociliary escalator. With increasing particle exposure, a focal collection of particle-laden macrophages results at the respiratory bronchiole (RB) which is that site in the clearance pathway demanding the greatest traverse by these cells after a commencement from the alveoli. With the greatest particle doses, there is “particle overload” and impaired mobility which is reflected by an excess accumulation of particle-laden macrophages throughout the RBs, alveolar ducts, and alveoli. With deposition of fibrous particles in the distal respiratory tract, the AM is unable to extend itself to enclose fibers with a major diameter of 10–20 microns or longer resulting in “frustrated phagocytosis” and longer retention times. Clearance pathways for particles are shared. There can be a summation of particle exposures with exhaustion in the capacity of the AMs for transport. Cigarette smoking (CS) is the greatest particle challenge humans encounter. Associated with its enormous magnitude, CS profoundly impacts the clearance pathways and subsequently interacts with other particle exposures to increase biological effects. Interstitial lung disease, pulmonary function, chronic obstructive pulmonary disease, infections, lung cancer, and mortality can be altered among smokers exposed to occupational and environmental particles (e.g., silica, coal mine dust, air pollution particles, other particles, and asbestos). It is concluded that both decreasing CS and controlling particle exposures are of vital importance in occupational and environmental lung disease.

## Introduction

The retention of occupational and environmental particles in the lung is a primary factor in determining their biological effects. Clearance pathways for particles are shared. Cigarette smoking (CS) is currently the greatest particle challenge humans encounter, by mass, with the respiratory tract exposed to between 15,000 and 40,000 μg/cigarette. The mean diameter of this particle is frequently <0.1 micron. Associated with its enormous magnitude, CS will profoundly impact the clearance pathways and subsequently interact with other particle exposures to biological effects.

### Particle clearance in the distal respiratory tract

**Figure 1:**
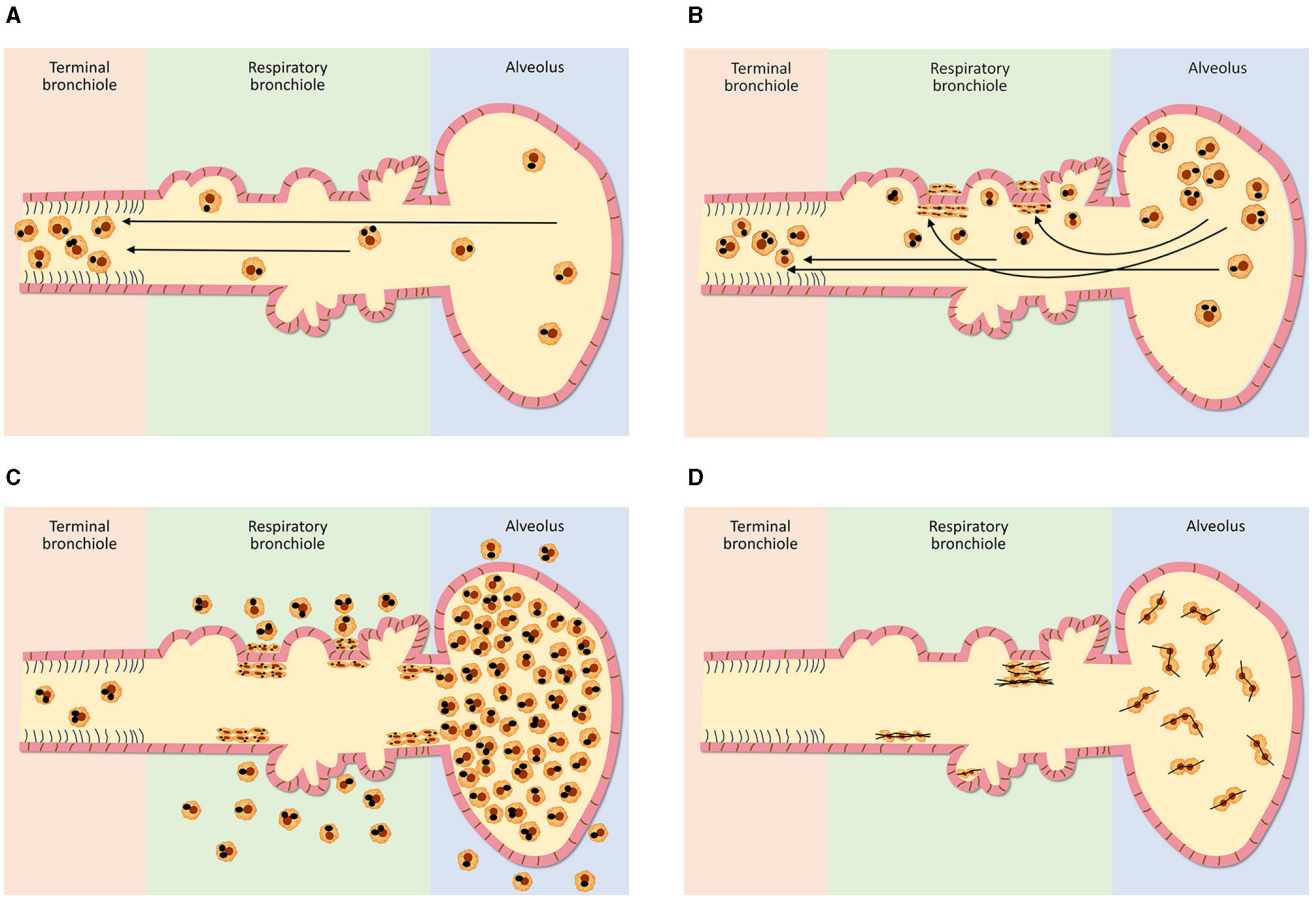
Schematics of clearance from the distal respiratory tract after varying doses of exposures to particles **(A–C)** and fibrous particles **(D)**. In the distal respiratory tract, particle clearance includes phagocytosis by alveolar macrophages, migration with the particle to the terminal bronchiole and transport of the macrophages, with the particle, by the mucociliary escalator **(A)**. The respiratory bronchiole is the most vulnerable site of this clearance pathway since it demands the greatest traverse of the macrophage and with increasing particle exposure, a focal collection of particle-laden macrophages (i.e., respiratory bronchiolitis) results here **(B)**. With the greatest particle doses, there is “particle overload” and impaired mobility of alveolar macrophages which is reflected by their excess accumulation in the respiratory bronchioles, alveolar ducts, and alveoli **(C)**. With deposition of fibrous particles in the distal respiratory tract, the AM is unable to extend itself to enclose fibers with a major diameter of 10–20 microns or longer **(D)**. Subsequently, there is diminished clearance with this “frustrated phagocytosis” resulting in long retention times.

The distribution of ventilation following air entry is affected by a gravitational gradient in the lung and, as a result, is 50% greater at the bases relative to the apices (1–3). Therefore, an increased number of particles is delivered to the lower relative to the upper lung fields. The retention of these particles is dependent on their incomplete clearance (4). The major pathway for clearance of particles deposited in the conducting airways is the mucociliary escalator which is both efficient and rapid (5). Ciliated epithelial cells which participate in this pathway continue to the terminal bronchiole (TB; sometimes designated the 16^th^ generation of airways) but do not extend into the distal airway. The respiratory bronchioles (RBs; sometimes designated the 17^th^ to 19^th^ generations of airways), alveolar ducts (ADs; sometimes designated 20^th^ to 22^nd^ generations of airways) and alveoli (i.e., the acinus) are removed from the mucociliary escalator (although the initial respiratory bronchioles can have some ciliated cells). In the acinus, particle clearance includes (1) phagocytosis by alveolar macrophages (AMs), (2) migration of these cells with the particle along the alveolar and bronchiolar surfaces to the TBs where the mucociliary removal mechanism begins, and (3) transport of the AMs by the moving surface fluid layer (6, 7) (Figure 1A). This clearance pathway is supported by the direct observation of “dust cells” (AMs with phagocytosed particles) on the intraluminal surfaces of airways (8). AM-mediated clearance is slower than that by the mucociliary escalator in the conducting airways and particle retention half-times in the distal respiratory tract are subsequently greater (9, 10). Eventually, the AMs with particle are either conveyed to the gastrointestinal tract via swallowing or expectoration (11).

Particle exposure stimulates a production of mediators recognized to accelerate monocyte release from the bone marrow (12). Blood monocytes accordingly increase in number after particle-associated exposures (e.g., CS, ambient air pollution, diesel exhaust, and traffic-related pollution) (13–17). This is not unique to lung exposures and an intraperitoneal injection of various particles (i.e., silica, kaolin, and polystyrene latex) can be associated with a comparable monocytosis in the peripheral blood (18). An accelerated release of monocytes from the bone marrow after particle inhalation is followed by their recruitment into the lung with differentiation to macrophages (12, 19). Particle exposure accordingly augments the numbers of macrophages recruited into the lungs allowing for their increased participation in clearance from the distal respiratory tract (19, 20).

The effects of gravity on the distribution of blood flow are attributed to the hydrostatic pressure difference between the top and bottom of the lung (2). At the uppermost parts of the lung, the pressure within the vessels may be less than the alveolar pressure, the vessels collapse, and the alveoli receive little to no blood flow (i.e., physiological dead space). In the lower lung zones, pulmonary venous pressure exceeds alveolar pressure. Therefore, perfusion is greatest in the lower lung fields comparable to ventilation (2). With a major source of alveolar macrophages being vascular monocytes, numbers of these cells present in the lung after particle exposure will accordingly be greatest in the lower fields reflecting perfusion (21). Based on a greater availability of monocyte-derived macrophages which phagocytose particles to expedite their removal, more proficient particle clearance in the lower lung fields is predicted.

**Figure 2:**
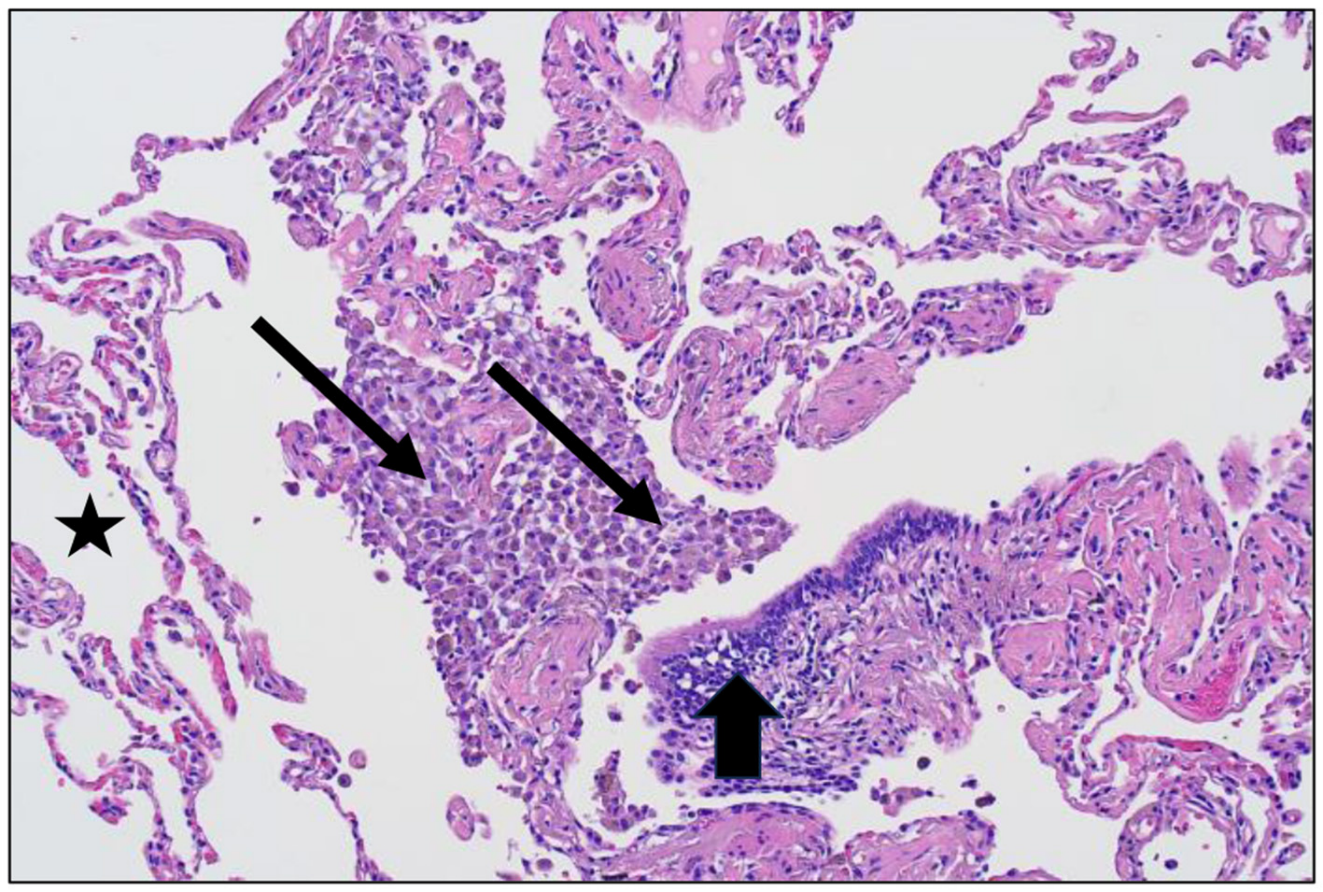
Lung tissue from a cigarette smoker demonstrating respiratory bronchiolitis after particle exposure (hematoxylin and eosin stain; magnification of approximately 100x). There is an accumulation of macrophages and pigmented particles evident at the respiratory bronchiole (designated by arrows) but not in the alveolar region (designated by a star) and the terminal bronchiole (designated by an arrowhead).

**Figure 3:**
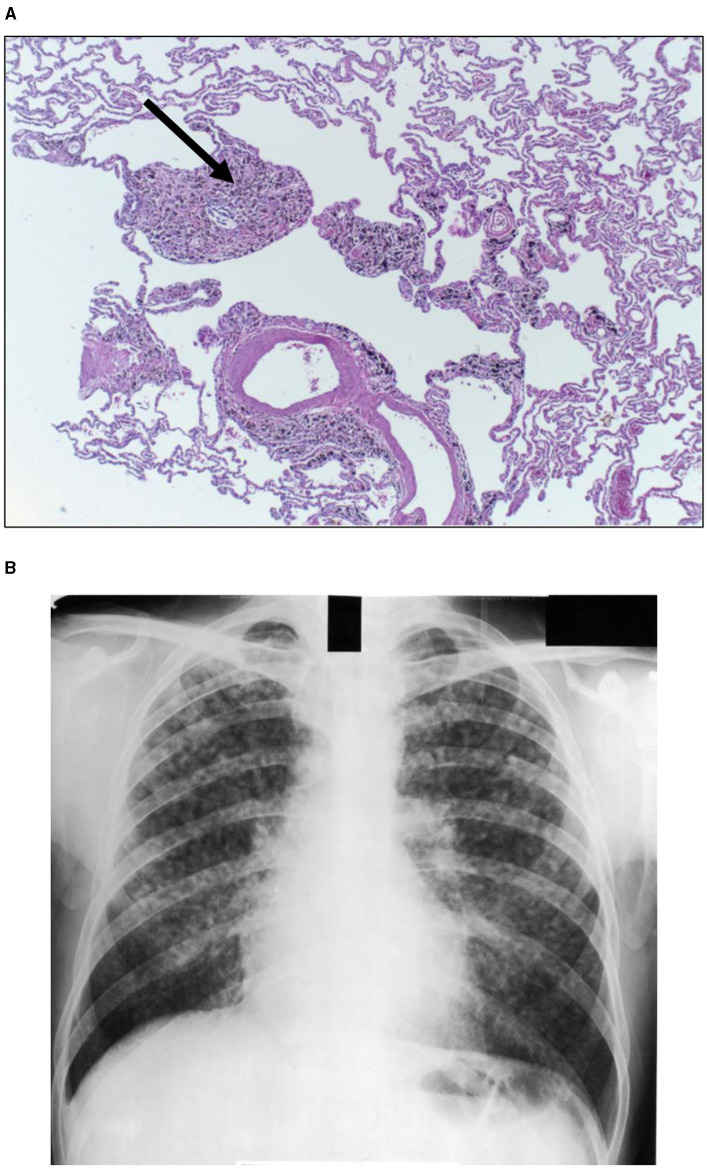
Human lung tissue from a coal miner reveals accumulation of particle in the region of the respiratory bronchiole recognized as a coal macule (designated by arrow) [**(A)**; hematoxylin and eosin stain; magnification of approximately 100x]. After accretion and coalescence, these collections of macrophages, particles, and associated inflammation and fibrosis can be observed as a simple pneumoconiosis on a chest X-ray **(B)**. Lesions are symmetrical involving both lungs and a preponderance in the upper lobes is noted.

The magnitude of exposure is a major factor which impacts particle clearance from the distal respiratory tract. As it increases, AM transport of particles from the surfaces of the RBs, ADs, and alveoli to the entry site of the mucociliary escalator (i.e., the TB) is overwhelmed (Figure 1B). The RB is that site in the clearance pathway demanding the greatest traverse by these cells originating in the alveoli and destined for the TB. There results a focal collection of particle-laden macrophages in the region of the RB (i.e., respiratory bronchiolitis; RBitis) following elevated particle exposures (Figure 2). The host response to phagocytosed particles will initially include inflammation but, when prolonged, there will be associated fibrosis with deposits of reticulin/collagen (22–24). When RBitis is associated with evidence of interstitial lung disease (ILD) including diffuse pulmonary infiltrates (typically patchy ground glass opacities on a CT scan) and/or pulmonary function impairment, the disease process is referred to as RB-associated interstitial lung disease (RB-ILD). These inflammatory and fibrotic lesions follow significant exposures most frequently in cigarette smokers but they can be associated with numerous different occupational and environmental particles (25, 26). The mass flow velocity into the airways approaches zero at the RB, augmenting mechanical deposition of particulate matter (27). It is at the level of the RB that maximal dust retention will occur with sheets of dust-laden macrophages which taper in number toward the level of the AD (21). With increased magnitude of the exposure, particles can further accumulate in the same region and histopathologic observations can include RBitis, macules, nodules, and simple pneumoconiosis (e.g., silicosis, coal workers' pneumoconiosis, kaolinosis, and talcosis) (28). Particle type will also affect the histopathology. Miners can show a high prevalence of simple pneumoconiosis, an exaggerated form of an RB-ILD with deposition of dust, macrophages, and connective tissue (Figure 3A) (29). The distribution of these lesions is symmetrical involving both lungs and most frequently with a preponderance in the upper lobes (Figure 3B). Toward the bases, these lesions tend to diminish in size and number. Such a conventional presentation of simple pneumoconiosis is consistent with particle clearance being most successful in the lower lung fields. These lesions can coalesce and while most often observed with silica and coal dust, the resultant large mass (i.e., complicated pneumoconiosis) can be observed after exposures to numerous, different particles. With the development of RBitis, macules, nodules, and simple pneumoconiosis, the clearance system is still effective in diminishing particle accumulation in the most distal respiratory tract where the exchange of oxygen and carbon dioxide occurs. Therefore, pulmonary function is usually normal in patients with simple silicosis and coal workers' pneumoconiosis and even with complicated disease.

**Figure 4:**
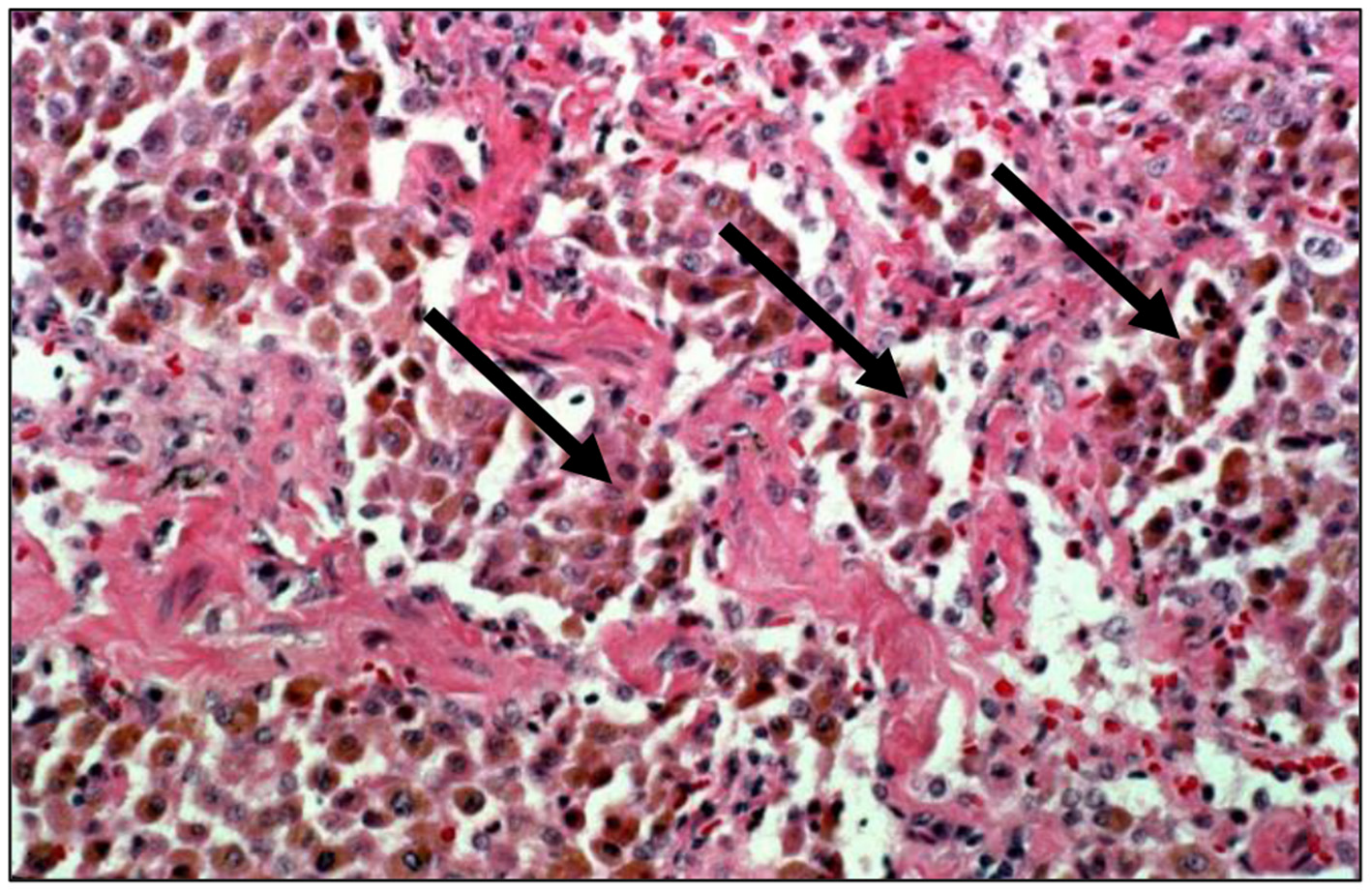
Human lung tissue from a smoker reveals an accumulation of macrophages in the alveolar region (hematoxylin and eosin stain; magnification of 100x). With extreme particle exposures, “particle overload” of the distal respiratory tract is reflected by an excess accumulation of particle-laden macrophages in the acinus (designated by arrows). This is pathologically recognized as desquamative interstitial pneumonitis. With smoking, the macrophages can contain fine brown granules supporting a failure of particle-laden macrophages to enter the mucociliary escalator.

With those particle exposures which are most extreme in magnitude, there can be a “particle overload” of the distal respiratory tract. With “particle overload,” there is a reduction in AM mobility associated with an impairment of clearance by these cells (30) (Figure 1C). Subsequently, the hallmark of the “particle-overloaded” lung is altered retention kinetics with the half-times increasing linearly with the burden (30–33). Changes in AM function with “particle overload” have been attributed to augmented particle mass, volume, and/or surface area (31, 32, 34–39). This is reflected by an excess accumulation of particle-laden macrophages frequently observed diffusely distributed throughout the RBs, ADs, and alveoli. These lesions become large, and irregular (often stellate). The histopathology is consistent with a desquamative interstitial pneumonitis (DIP) which is defined to include an alveolar accumulation of particle-laden macrophages (Figure 4). This can be observed with CS, passive smoking, and other particles and are associated with interstitial inflammation and fibrosis (40).

An inability of the AMs to eliminate particles from the distal respiratory tract recruits alternative routes or pathways of clearance. The accumulation of AMs in the distal respiratory tract accelerates their migration with phagocytosized particles into the interstitium and subsequent transport to lymphatics, lymph nodes, and pleura (41). Particle-laden AMs penetrate the pulmonary interstitium (8, 41). AMs with phagocytosed particles then accumulate in bronchus-associated lymphoid tissue, migrate to peribronchial and perivascular lymphatics, and are transported (1) antegrade to regional lymph nodes (41–43) and (2) retrograde to the pleura (44). In animal models, AMs with phagocytosed particles can be identified in the pulmonary interstitium (45, 46), intravascular and sub-epithelial locations in the bronchioli (47), and in draining lymph nodes (45, 48). Particle numbers increase in the thoracic and retroperitoneal lymph nodes, accordingly, these appear black in smokers and coal miners (49). The lymphatics also transport particles retrograde to sub-pleural areas with access to the pleural space. While there are no direct connections with the lung, specific areas of the parietal pleura absorb and retain high concentrations of particles from the pleural space. These particle-collecting structures have been called “black spots” which can be observed in 92.7% of urban autopsies (50, 51). “Black spots” of the pleura develop in close correlation to lymphatic channels and blood vessels. AMs accordingly can redistribute a particle burden across the epithelium into the interstitium, lymphatics, lymph nodes, and pleura (6, 41). Inflammation and fibrosis can result wherever the particle is retained. In addition to pneumoconiosis in the lung parenchyma, inflammation and fibrosis can also impact the lymph nodes and the pleura. With particle exposures, lymphadenopathy with both inflammation and fibrosis is observed (42, 52–58). In the pleura, “black spots” are characterized by an inflammatory reaction to the incorporated particles and a fibrosis comparable to lymph nodes (59–61). Increased particle burdens at “black spots” following occupational exposures to mixed dusts may affect macrophage function (i.e., transport and phagocytosis) (59).

### Cigarette smoking and particle clearance

The biological effects after smoking can be related to particle exposure (62–65). The mean diameter of CS particles is 0.2–0.5 μm and the deposition fraction is projected to be 70%−90% with the greatest of this occurring at the RBs (66, 67). CS accelerates a release of monocytes from the bone marrow and these are recruited to differentiate to macrophages accounting for increased numbers in the smoker's lung and allowing for their participation in clearance from the distal respiratory tract (12, 19, 21). Despite this, particle clearance was demonstrated to be impaired in smokers with 50% retention compared to 10% in non-smokers (68). Exposure to CS impacted mucociliary clearance with decreases in both ciliated cell numbers and ciliary beat frequency (69). Accordingly, CS decreases the efficiency of clearance from both the alveolar and bronchial compartments and smokers retain more particles than non-smokers (70).

CS is the paramount particle exposure and it is frequently associated with a “particle overload.” Since AMs normally direct the clearance process in the distal respiratory tract, pathways are overwhelmed with CS and particles are not successfully removed from the RBs, ADs, and alveoli; particle retention results (30). Histopathologically, smoking is characteristically by an accumulation of AMs containing fine brown granules which do not polarize light in the distal respiratory tract (Figure 4). There can be varying amounts of fibrosis in smoking-related RBitis, RB-ILD, and DIP (71). RBitis is a universal response in all smokers (i.e., smoker's bronchiolitis) characterized histologically by a patchy accumulation of pigmented, smoker's macrophages in the RB (72–75). This reflects the failure of particle-laden AMs to enter the mucociliary escalator at the TB. Loss of epithelial ciliation as well as alterations in lung architecture such as observed in chronic obstructive pulmonary disease (COPD), as well as other chronic lung diseases after long-term smoking can further particle retention. With continued smoking, greater particle deposition initiates an inflammatory and fibrotic response consistent with DIP; CS is associated with most DIP cases (76, 77). While the feature that differentiates RBitis from DIP is most frequently cited as the distribution and extent of macrophage accumulation (bronchiolocentric in RBitis and diffuse throughout the entirety of the RBs, ADs, and alveoli in DIP), there are no reliable histologic features to distinguish the two responses with complete certainty and they can be considered separate phases of a single response (78). Smoking cessation is currently considered the primary treatment for both RBitis and DIP and survival rates are high. This inflammatory response can persist after smoking cessation as CS particle persists in the lung for years (79–82).

With an absence of effective clearance pathways associated with “particle overload,” the biological response (inflammation and fibrosis) with CS will be evident in the lower lung fields where the ventilation, and exposure, is greater relative to the upper lung fields. Subsequently, radiographic evidence of smoking is recognized to include opacities in the lower lung fields reflecting the inflammation and fibrosis in response to CS particle (83–89). In the last two decades, interstitial lung abnormalities (ILAs) have been observed on 8%−20% of smokers' CT scans screened for lung cancer and without clinical symptoms (90–95). On pathological examination, ILAs include both inflammation and fibrosis (95, 96). These ILAs are a precursor to clinically evident smoking-related lung fibrosis.

Evidence supports a causative relationship between smoking and lung inflammatory and fibrotic disease (72, 77, 82, 97–103). Before chest X-rays and CT scans of the chest demonstrate any abnormalities, inflammation and fibrosis can be observed histologically in most smokers (96). This chronic pulmonary inflammation and fibrosis can progress and manifests as several different histologic patterns: (1) organizing pneumonia (OP), characterized by round or oval pale-staining deposits consisting of fibroblasts, myofibroblasts, collagen, and fibrin within RBs, alveolar ducts and alveoli; (2) nonspecific interstitial pneumonia (NSIP), characterized by inflammation and/or fibrosis in the lung interstitium occurring in a spatially homogeneous pattern and with preservation of overall lung architecture; and (3) usual interstitial pneumonia (UIP), the most severe form of lung fibrosis, characterized by heterogeneous areas of dense fibrosis interspersed with areas of relatively normal lung architecture, fibroblastic foci, and honeycombing (82, 97, 101, 104–111). OP and NSIP can represent earlier phases of the response to CS while UIP represents a response to greater doses (pack-years) and subsequently a later stage. One histologic presentation of injury may progress into the next with overlapping patterns being observed which make strict histopathologic diagnosis difficult or impossible (112, 113). OP components are common lesions in both NSIP and UIP cases, and as fibrosis evolves, pathological areas of NSIP are observed with UIP (114–119). It is the retention of particle in the distal respiratory tract with CS which is proposed to impact a dose-response fibrosis (120).

In smokers, excessive accumulation of AMs accelerates their “interstitialization” and migration into lymphatics, lymph nodes, and pleura. With the biological effects after smoking being related to particle exposure, inflammation and fibrosis then occur in the interstitium, bronchus-associated lymphoid tissue, peribronchial and perivascular lymphatics, regional lymph nodes, and pleura as well as the distal respiratory tract. Enlarged mediastinal lymph nodes occur in over 50% of heavy smokers (43).

### Smoking and disease after exposure to silica

Comparable to all particles, the clearance of silica in the distal respiratory tract includes phagocytosis by AMs with transport to mucociliary escalator at the TB. With exposure to silica, there can be an RBitis which progresses to RB-ILD and pneumoconiosis (simple and complicated). In silicosis, the upper lobes reveal greater involvement since particle clearance is more effective at the bases. With greater exposures to silica, AM mobility will be impaired and the particle may not be successfully translocated to the TB. This sequence suggests a dose-response in which there is an initial increase in the accumulation of particles removed from the distal respiratory tract to the region of the RB (i.e., pneumoconiosis) followed by patterns of involvement reflecting an injury including DIP; idiopathic cases of DIP demonstrate a history of particle exposure including silica (121). In addition, alternative routes or pathways of clearance (e.g., interstitium, lymphatics, lymph nodes, and pleura) are recruited at higher exposure levels and other forms of inflammatory and fibrotic lung disease can be evident (e.g., OP, NSIP, and UIP as well as sarcoidosis) (122, 123).

Collagen deposition and fibrosis increase with particle retention in the lung and the failure of clearance pathways including alveolar macrophages to remove particle from the distal respiratory tract. A dose response of fibrotic lung injury with several different particles has been demonstrated (e.g., silica, coal mine dust, and asbestos) (124). Dissimilarities between dose-response of these particles is also evident (e.g., silica generates a greater fibrotic response relative to coal mine dust).

Interactions are described between smoking and silica exposure and these can vary widely depending on the specific endpoint (Table 1) (125). Smoking increases the total particle delivered to the distal respiratory tract and clearance mechanisms are subsequently overwhelmed. Smokers exposed to silica dust have been observed to develop silicosis more frequently than non-smokers exposed to the same dose (126–129). In one study, 85% of silicosis cases were ever smokers (vs. 70% in controls) (130). In the pottery and stone benchtop industries, silicosis was associated with smoking (131, 132). In these investigations, the types of opacities observed on the chest X-ray were not specified while others analyzed (1) only rounded opacities consistent with conventional silicosis or (2) both rounded and irregular opacities. Many of the opacities in silicosis among smokers were irregular and observed in the lower lung fields which is not considered consistent with the appearance of conventional silicosis (133, 134). Animal studies support an interaction between smoking and silica with greater biological effect initiating lung inflammation and fibrosis after combined exposures (135).

**Table 1:** Interactions of silica with smoking.

	**Impact of smoking**
Interstitial lung disease	Increased silicosis
	Decreased silicosis
	Changes silicosis to include irregular opacities
Pulmonary function tests	Excess loss in spirometry indices
COPD	Higher rates
Infections	Increased tuberculosis rates
Malignancy	Increased lung cancer
Mortality	Increased overall mortality
	Increased lung cancer mortality

With a summation of particles in CS and silica exposure, there is an exhaustion in the capacity of the AMs for transport. Subsequently, the pattern of biological effect can change. In smokers, exposure to silica may not be associated with nodules and rounded opacities in the upper lung fields. Rather, opacities after silica exposure in smokers may be in the lower lung fields where most of the inhaled breath with particles is distributed. While smoking can increase incidence of silicosis, it might also alter the natural history of silicosis. In support of this, a slight inverse relationship between smoking and collagenization of the parenchyma was observed (after controlling for age and cumulative exposure to silica dust) among deceased miners who underwent postmortem examination between 1976 and 1981 (136). While smoking could decrease the incidence of pneumoconiosis, the silica would accumulate in different sites; these would be more distal and contribute to other types of inflammation and fibrosis (e.g., OP, NSIP, and UIP). Such patterns of fibrotic lung injury have been observed after silica exposure and almost all were among smokers (137–139).

Like numerous particle exposures, silica is associated with pulmonary function loss (140). The estimated excess loss of lung function for a 50-year-old gold miner exposed to silica associated with 24 years of underground dust exposure at an average respirable dust concentration of 300 μg/m^3^ was 236 mL of forced expiratory volume in 1 second (FEV_1_) and 217 mL of FVC (forced vital capacity) (141). By comparison, the effect of smoking one packet of cigarettes a day over 30 years was associated with an estimated loss of 552 mL of FEV_1_ and 335 mL of FVC in this same study. Interactions between smoking and silica exposure can be observed with potentiation of the effect of silica on pulmonary function loss, rates of airway obstruction, and prevalence of COPD (142–146). CS was found to potentiate the effect of dust, predominantly silica, on respiratory impairment in gold miners (147). Those with silicosis had greater rates of COPD with smoking (148). The two particles also interacted to impact the capacity of AMs to eliminate respiratory pathogens in workers substantiating a critical role for these cells in the prevention of infection (149, 150). Current smokers had a significantly higher risk of tuberculosis than other patients with silicosis (125, 151, 152). A negative relationship between CS and collagenization of the pleura was observed among deceased miners on postmortem examination suggesting an impact of smoking on lymphatic clearance to the pleura (136). While there is a dose-response between the cumulative exposure to silica and lung cancer among never-smokers, 91% of silica-related lung cancer cases were ever smokers (vs. 69% controls) (130, 153–155). The joint effects of smoking and silica on lung cancer incidence have been additive, super-additive, multiplicative, and even supra-multiplicative (153, 156). Excess lung cancer mortality in silica-exposed workers was restricted to those with silicosis, and the more severe the disease, the higher the neoplastic risk (153, 157, 158). One investigation demonstrated that most of the excess lung cancer risk in patients with silicosis was due to smoking, but a synergistic effect between smoking and silica/silicosis was likely (159). Finally, smoking increases mortality following silica exposures with interactions between silica and smoking increasing deaths after pulmonary diseases including pneumoconiosis and COPD (142, 143, 160, 161).

### Smoking and disease after exposure to coal mine dust

The fundamental lesion of coal workers' pneumoconiosis consists of a focal aggregation of coal mine dust (CMD)-laden AMs that accumulate in the RB and TB (28). This collection of dust-laden AMs, reticulin and collagen (1–4 mm in diameter) is referred to as a coal dust macule; these are non-palpable and distributed throughout the lung with greater numbers in the upper lung fields (162). The coal macule is a RBitis. With increased exposure to CMD, AMs with particles accumulate to extend into adjacent regions as well as into the peribronchiolar interstitium, the size of the coal macule increases (some by accretion until they become visible whereas others coalesce with adjacent nodules to become macroscopically visible), and these eventually become fibrotic nodules of simple coal workers' pneumoconiosis (29). This simple pneumoconiosis demonstrates a centrilobular (centered around the RB) deposition of coal dust, AMs, inflammation, and connective tissue. Complicated lesions of coal workers' pneumoconiosis (defined pathologically and radiologically as nodules measuring 1 cm or more in diameter) follow the fusion of small opacities, an accretion and incorporation of adjacent nodules. Mediastinal and hilar adenopathy can be present but this is usually in less than one-third of the cases. Exposure to CMD can cause DIP with particle-laden (anthracotic and silica/silicate) AMs accumulated in the distal respiratory tract (163). This advances to o types of inflammation and fibrosis (e.g., OP, NSIP, and UIP). Cases of a UIP pattern of lung fibrosis with honeycombing on the CT scan was reported among coal miners (pathology was available in 2 explanted lungs and 6 open lung biopsies) (164).

The research into interactions between coal mine dust and smoking has been limited (Table 2). Early investigation suggested no influence of smoking on the coal dust macule and its direct complications; coal miners who did smoke had a greater degree of corpulmonale, more emphysema, and more bronchiolar goblet cells with chronic bronchiolitis than non-smoking coal miners (165). Coal miners with primarily irregular opacities showed a lower zone preponderance (166). In investigation, irregular opacities on radiographs of South Wales coal workers were related to age, smoking and coal work exposure (167). Similarly, irregular small opacities on the radiographs of coal miners in the USA were associated with smoking, age, years worked underground, and a diagnosis of bronchitis (168). There is a description of chronic interstitial pneumonia with honeycombing in coal miners (164). Among the 38 miners included in this report, 32 were smokers. Dust-related diffuse fibrosis was described recently in a significant number of autopsies of coal workers (15% to 20%) (169, 170). Dust-related diffuse fibrosis can resemble idiopathic pulmonary fibrosis (IPF), which is associated with smoking, but has a better prognosis. 44/45 of miners with diffuse ILD were smokers.

**Table 2:** Interactions of coal mine dust with smoking.

	**Impact of smoking on endpoint**
Interstitial lung disease	Changes coal workers' pneumoconiosis to include irregular opacities
Pulmonary function tests	Excess loss in spirometry indices
COPD	Higher rates
Mortality	Increased mortality (among those with complicated disease)

An interaction between smoking and CMD on pulmonary function has been demonstrated. Combined effects of smoking and CMD exposure on pulmonary function were significantly greater than that of the CS only and cumulative total exposure only (171). CMD positively correlated with abnormal rates of pulmonary function in both the smoking and non-smoking subgroups; one index (i.e., the FEV_1_) was negatively correlated with total dose only in the smoking subgroup (172). Spirometric indices (i.e., forced expiratory volume in one second and the ratio) were lower among those coal miners with irregular opacities possibly reflecting CS (168). Miners with coal workers' pneumoconiosis demonstrated greater rates of COPD with cigarette smoking (148). While there is little evidence of a CMD exposure-lung cancer risk, lung nodules among smokers with coal workers' pneumoconiosis had a higher risk of being lung cancer (173, 174). Finally, smoking increased mortality of those coal miners with complicated pneumoconiosis (175).

### Smoking and disease after exposure to air pollution particles

Ambient air pollution particles are retained in the human lung with greater concentrations (number counts) observed in the RBs which accumulated high particle loads, typically 25–100 times the concentrations seen in the mainstem bronchus (176). The mean diameter of this air pollution particle was 0.38 micron. Using analytical electron microscopy, particles retained in autopsy lung tissue from 10 never-smokers were shown to include silica, silicates (kaolin, feldspar, mica, and talc), and metals (176, 177); the majority of these particles appeared to be crustal in origin. However, examination of human lungs confirmed that airways could also retain relatively large numbers of carbonaceous chain aggregates of particles (ultrafine in size) that appeared to be combustion products (178). Black pigmented particles accumulated in the non-smoker's lung with increasing age and this was attributable to soot in the ambient air originating from the burning of coal and wood (179). Emission source air pollution particle (e.g., environmental tobacco smoke) was associated with a RBitis comparable to other particles (25, 26). Air pollution particles were also observed in the peribronchial lymph nodes (forming “black glands”) (179). Disease after air pollution particles included a diffuse pulmonary fibrosis with exposures to combustion products of biomass fuels used for cooking and heating in poorly ventilated huts (180).

There was an interaction between air pollution and smoking observed in decreasing pulmonary function (181) (Table 3). Synergistic interactions between air pollution, specifically including particles, and smoking in contributing to cardiovascular mortality were observed in additional epidemiological investigation (182, 183). In smokers, there was a significant excess risk of daily mortality associated with ambient air pollution particles for all natural causes and cardio-respiratory diseases for men aged 30 years or older and men 65 or older (184). Mortality risks caused by all natural causes and cardiorespiratory diseases per 10 μg/m^3^ change in black smoke were higher in current smokers than never smokers (185). Ever-smokers were again more susceptible to excess mortality risk associated with daily air pollution (186).

**Table 3:** Interactions of air pollution particles with smoking.

	**Impact of smoking on endpoint**
Pulmonary function tests	Excess loss in spirometry indices
Mortality	Increased cardiovascular mortality
	Increased cardio-respiratory mortality
	Increased overall mortality

### Smoking and disease after exposure to other particles

Vermiculite and smoking interact to increase the interstitial markings on the chest X-rays of miners (187). The prevalence of opacities on the chest radiographs of talc millers could similarly be increased among smokers (188). Flour dust also interacts with smoking to impact an ILD observed on the CT scan (189). Regarding pulmonary function testing, the effects of cotton dust exposure and cigarette smoking on spirometry endpoints appeared to be additive (190–192).

### Asbestos clearance in the distal respiratory tract

Fibrous morphology has been identified as a major characteristic in determining clearance (193). With deposition in the distal respiratory tract, the AM is unable to extend itself to enclose longer fibers (those with a major diameter of 10–20 microns or longer) resulting in incomplete or “frustrated phagocytosis”, long retention times, and persistent biological effect (e.g., inflammation, fibrosis, and neoplasia) (194) (Figure 1D). Therefore, fiber dimensions are a major determinant in the biological effect of asbestos (195). The risk for biological effect of asbestos correlates with fiber length (196, 197). Asbestos relevant to fibrosis and cancer can include those longer than 5 microns (198). Short fibers (those < 5 μm) are fully engulfed (internalized) by macrophages and removed by lung clearance more efficiently (193, 199–202). Accordingly, while short fibers may also be associated with biological effects, these are observed most frequently only at greatly increased exposure levels (203, 204).

**Figure 5:**
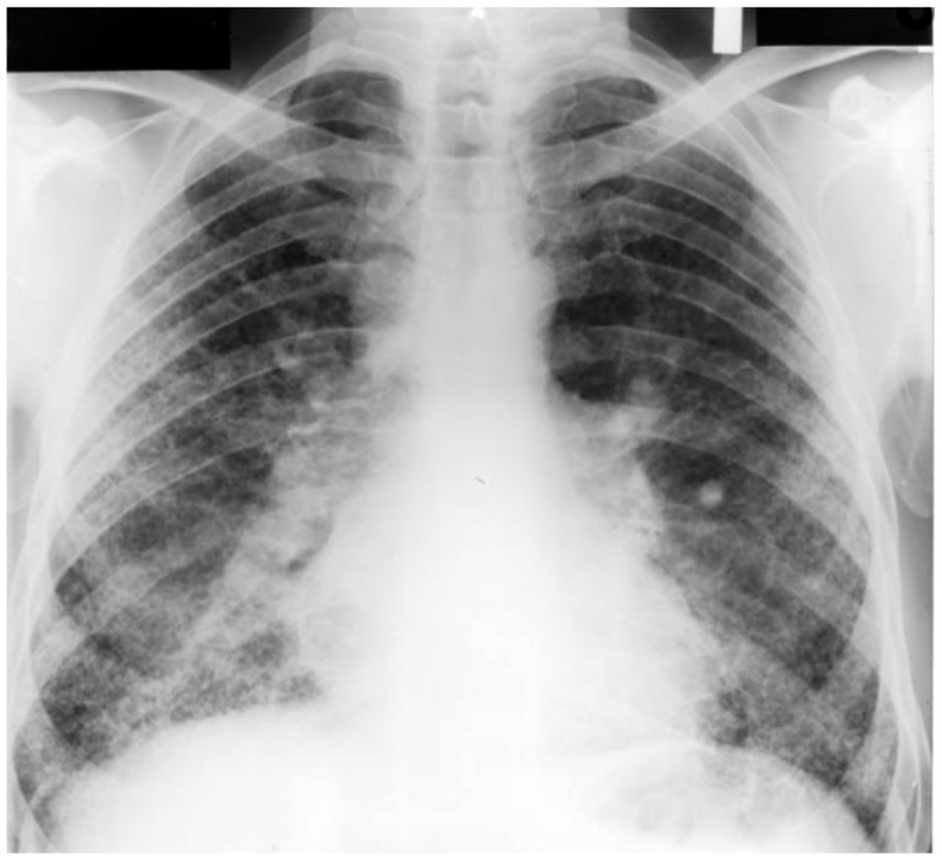
A chest radiograph of inflammatory and fibrotic injury following asbestos exposure. This is characterized by bilateral, small irregular opacities involving the lower lobes of the lungs. The dimensions of asbestos preclude efficient clearance (“frustrated phagocytosis”). Subsequently, in contrast to particles, the associated inflammation and fibrosis is in the lower lung fields where delivery is greater relative to the upper lung fields.

Asbestos exposure is associated with both RBitis and DIP (205–207). With ineffective clearance, disease associated with asbestos occurs at much lower levels of exposure relative to other particles such as silica and coal (208). Subsequently, the exposure (the dose of dust retained in the lungs) is far less in asbestosis (i.e., milligrams) relative to silicosis and coal workers' pneumoconiosis (i.e., grams or tens of grams) (209, 210). In addition, the radiographic and pathologic appearance of asbestosis are distinct from other pneumoconioses associated with particle exposure (e.g., silicosis and coal workers' pneumoconiosis). Asbestosis is characterized by the presence of small irregular opacities, which are bilateral and commonly involve the lower lobes of the lungs (205, 211–216) (Figure 5). This presentation is the result of the particle being distributed to the lower lung fields and an ineffective transport from the distal respiratory tract to the entry site of the mucociliary escalator (i.e., the TB). For this same reason, asbestos exposure is not associated with complicated pneumoconiosis.

The distribution of mineral fibers in the lung determines the site and severity of disease (217). With decreased mobility of the AM following “frustrated phagocytosis,” asbestos is translocated to the interstitium where it is transported via lymphatics antegrade to the lymph nodes and retrograde to the pleura and the peritoneum (218, 219). As a result, thoracic lymph node enlargement occurs frequently with asbestos exposure (220). From the lungs, asbestos fibers can migrate to pleural and peritoneal spaces following patterns of lymphatic drainage. In addition, a proportion of asbestos that reaches the pleura will pass through the pleural space and exit through the stomata. Subsequently, the fibers are sequestered in the “black spots” in the parietal pleura. Fibers that reach the pleural space can interrupt flow with interception of the asbestos within the walls of the stomatal openings as well as in the lymph vessels walls. This increases an accumulation of pleural macrophages attempting to phagocytose retained, long fibers. The most frequently documented pleural response to fibers are effusions (i.e., inflammation) and plaques (i.e., fibrosis). The latter are dense bands or weaves of collagen, frequently calcified, and occur on the parietal pleura at sites where the stomata are in greatest profusion. The pleural response is observed with numerous particle exposures with the severity being dependent on the biological activity of the dust (i.e., carbonaceous < mixed mineral dust < silica < short asbestos < long asbestos). Pleural plaques are a special case of a “black spot” caused by fibers eliciting an extreme collagenous response. Finally, there is a risk of cancer (mesothelioma and lung cancer in the pleura and lung respectively) among workers exposed to asbestos which increases with exposure to longer fibers (200, 221–224). Animal investigation supports that longer fibers (>10 μm) are more carcinogenic to the lung (221).

### Smoking and disease after exposure to asbestos

There is an interaction between smoking and asbestos exposure to impact an increased incidence of small opacities observed on both the chest X-ray and CT scan as well as in pathological specimens (89, 225–227) (Table 4). A dose-response pattern emerged between increasing pack-years and parenchymal opacities (228). In those reports where the type of interaction has been addressed, the data in general have favored an additive effect but this could be greater (93, 221). In the US Navy's Asbestos Medical Surveillance Program, the prevalence of definite radiologic parenchymal abnormalities (ILO category ≥ 1/0) was 3.10 percent for 32,233 smokers vs. 1.09 percent for 13,414 non-smokers. Mechanistic endpoints also support interactions between smoking and asbestos exposure in lavage cellularity in patients with asbestosis (229). However, increases in profusion of asbestosis with smoking have been attributed to diffuse interstitial fibrosis (88, 213). Smoking can also increase the risk for pleural thickening/plaques (230–234). Plaques in ever smokers are proposed to result with clearance pathways decreased by smoking leading to higher exposures of the pleura.

**Table 4:** Interactions of asbestos with smoking.

	**Impact of smoking on endpoint**
Interstitial lung disease	Increased asbestosis
Pleural disease	Increased pleural plaques
Pulmonary function tests	Excess loss in spirometry indices and diffusing capacity
Malignancy	Increased lung cancer

Reduction in both FVC and FEV_1_/FVC were more frequent in insulators who smoked compared with non-smokers or smokers in the general population supporting an interaction (235, 236). Occupational exposure to asbestos in the cement industry was a risk factor for increased lung function decline which synergistically interacted with smoking (237). There was an interaction between asbestos exposure and smoking in the impact on pulmonary function (238, 239). Additive but not synergistic effects between exposures to smoking and asbestos were present for manifestations of asbestosis including bilateral fine crackles, clubbing, dyspnea, radiographic abnormality, decreased forced vital capacity, and decreased single breath diffusing capacity of the lung for carbon monoxide (240).

Finally, an interaction between smoking and exposure to asbestos with lung cancer risk was confirmed (241–250). This can range from additive to supra-additive to multiplicative.

## Conclusions

There is an interaction between smoking and other particles which impacts the biological effects of occupational and environmental exposures. This interaction is mediated by an impact of smoking on clearance. Smoking will almost always increase the risk for or exacerbate many particle-related lung diseases. In occupational and environmental lung disease, both decreasing CS and controlling particle exposures are of vital importance (251–253).
